# One-night sleep deprivation induces changes in the DNA methylation and serum activity indices of stearoyl-CoA desaturase in young healthy men

**DOI:** 10.1186/s12944-016-0309-1

**Published:** 2016-08-26

**Authors:** Gudrun Valgerdur Skuladottir, Emil Karl Nilsson, Jessica Mwinyi, Helgi Birgir Schiöth

**Affiliations:** 1 Department of Physiology, Faculty of Medicine, University of Iceland, Vatnsmyrarvegur 16, IS-101 Reykjavik, Iceland; 2 Department of Neuroscience, Functional Pharmacology, Uppsala University, Uppsala, Sweden

**Keywords:** DNA methylation, Fatty acid composition, Monounsaturated fatty acids, Sleep condition, Stearoyl-CoA desaturase

## Abstract

**Background:**

Sleep deprivation has been associated with obesity among adults, and accumulating data suggests that stearoyl-CoA desaturase 1 (SCD1) expression has a relevant impact on fatty acid (FA) composition of lipid pools and obesity. The aim of this study was to investigate the effect of one-night total sleep deprivation (TSD) on DNA methylation in the 5’-prime region of SCD1, and whether detected changes in DNA methylation are associated with SCD activity indices (product to precursor FA ratios; 16:1n-7/16:0 and 18:1n-9/18:0) derived from serum phospholipids (PL).

**Methods:**

Sixteen young, normal-weight, healthy men completed two study sessions, one with one-night TSD and one with one-night normal sleep (NS). Sleep quality and length was assessed by polysomnography, and consisted of electroencephalography, electrooculography, and electromyography. Fasting whole blood samples were collected on the subsequent morning for analysis of DNA methylation and FAs in serum PL. Linear regression analyses were performed to assess the association between changes in DNA methylation and SCD activity indices.

**Results:**

Three CpG sites close to the transcription start site (TSS) of SCD1 (cg00954566, cg24503796, cg14089512) were significantly differentially methylated in dependency of sleep duration (−log_10_*P*-value > 1.3). Both SCD-16 and SCD-18 activity indices were significantly elevated (*P* < 0.05) following one-night TSD, and significantly associated with DNA methylation changes of the three mentioned probes in the 5’ region of SCD1.

**Conclusion:**

Our results suggest a relevant link between TSD, hepatic SCD1 expression and de-novo fatty acid synthesis via epigenetically driven regulatory mechanisms.

## Background

Sleep deprivation has been associated with higher risk of weight gain and development of obesity among children and adults [1–3], which may provoke a higher susceptibility to chronic illnesses, such as diabetes [4, 5], and cardiovascular diseases [6, 7]. Human studies have demonstrated that sleep deprivation alters the central nervous system driven control of both hunger and appetite, provoking excessive food intake [8–10]. However, the knowledge of the complex and multifactorial mechanisms between sleep duration and increased risk of weight gain and obesity is still limited.

A recently published study has demonstrated that rhythmic expression patterns of clock and selected clock-controlled genes in human blood cells are in part determined by exogenous factors, such as sleep and fasting state, and in part by the endogenous circadian timing system [11]. Furthermore, new results indicate that acute sleep loss alters the epigenetic and transcriptional profile of core circadian clock genes in key metabolic tissues [12], and that longer habitual sleep duration could ameliorate genetic predisposition to obesity via a favorable dietary profile [13].

The enzyme stearoyl-CoA desaturase (SCD), which is predominantly expressed in the liver, plays a central role in the desaturation of saturated fatty acids (FAs), thus having important implications in the metabolism of FAs and development of obesity. SCD catalyzes the biosynthesis of the monounsaturated FAs palmitoleate (16:1n-7) and oleate (18:1n-9) from the saturated FAs palmitate (16:0) and stearate (18:0), respectively [14]. In sated state the FAs of phospholipids (PL) and triglycerides (TG) in healthy liver originate from nonesterified FAs, dietary FAs incorporated in chylomicrons and FAs synthesized by hepatic de novo lipogenesis from dietary carbohydrates [15–17]. The de-novo FA synthesis in liver is well reflected by SCD activity indices estimated as ratios of 16:1n-7/16:0 and 18:1n-9/18:0 in blood TG as well as in PL [18–21]. Over the last decade there has been much interest in estimating the SCD activity as a putative biomarker for body fat regulation and development of obesity.

Studies have shown that there is a very tight and complex regulation of SCD1 gene expression in response to various parameters including hormonal and nutrient factors [22, 23]. Furthermore, elevated expression levels of the human SCD1 gene are found to correlate both with the SCD enzyme activity [24], and obesity [25].

Recently, it has been demonstrated in healthy subjects, who have been also included in the current study that one-night of total sleep deprivation (TSD) alters clock gene regulation, concomitant with deleterious metabolic effects, which are differential across key peripheral metabolic tissues in healthy humans [12]. Hitherto, no study has addressed the effect of sleep deprivation on cytosine DNA methylation of SCD1 that might have implications for SCD activity, endogenous lipid biosynthesis and development of obesity. Therefore, we assessed DNA methylation of selected CpG sites within the SCD1 promoter and studied the associations with SCD activity indices derived from serum PL following both one-night normal sleep (NS) and one-night TSD in young healthy men.

## Methods

### Study cohort and design

This study was based on prospectively collected data from a randomized crossover within-subject trial designed to examine the effects of one-night TSD on gene expression and DNA methylation of core circadian clock genes in peripheral tissues. The study was registered with ClinicalTrials.gov, number NCT01730742. Details of the study design have been published previously [12]. Sixteen young (age: 23.3 ± 3.4 (mean ± S.D.) years), normal-weight (BMI: 23.64 ± 2.35 kg/m^2^), and healthy men participated in two sessions of this study. In brief, on day 1 the participants were provided standardized meals including breakfast, lunch, snack, and dinner. The next night nocturnal sleep was either permitted from 22:30 h (lights off) to 06:30 h (lights on; one-night NS) or the participants remained exposed to light under constant supervision from 22:30 h to 06:30 h, remaining bed-restricted and fasted, i.e. one-night TSD. All participants were engaged in both conditions using a within-subject, randomized crossover design, where each condition was separated by 4 weeks. Written, informed consent was obtained from all participants and the regional ethical committee in Uppsala, Sweden approved the study. Sleep quality and length was assessed by polysomnography (Embla A10, Flaga hf, Reykjavik, Iceland), and consisted of electroencephalography, electrooculography, and electromyography. Blood samples were collected in EDTA tubes in the evening (19:30 h) of day 1, and in the morning after the intervention and before a standardized breakfast (07:30 h) of day 2. Fasting whole blood samples for DNA methylation analysis were immediately frozen in a 50/50 mixture of ethanol and dry ice before deposited in −80 °C. Serum samples were separated from fasting whole blood and stored in −80 °C before FA analysis.

### Sample preparation and methylation analyses

Genomic DNA was extracted by robot assisted phenol/chloroform extraction at the Latvian Biomedical Research and Study Centre in Riga, Latvia. Bisulfite conversion of DNA and hybridization to the Illumina 450 K methylation Bead Chip (Illumina, San Diego, USA) was performed at the Science for Life Laboratory (SciLifeLab Uppsala, Sweden). Beta values representing the methylation status (0–100 %) of SCD1, localized on chromosome 10, were generated by GenomeStudio (Illumina, San Diego, USA).

### Determination of SCD-16 and SCD-18 activity

The total serum lipid fraction was extracted with chloroform-methanol (2:1, v ⁄v), using a well-established method [26]. PL were separated on a TLC plate using the solvent system petroleum ether⁄diethyl ether ⁄acetic acid (80:20:1, v⁄v⁄v). The PL FAs were methylated with 14 % boron trifluoride in methanol, and the FA methyl esters analyzed by a HP Series II 5890, series A Gas Chromatograph (Hewlett Packard Co⁄Agilent, Palo Alto, CA, USA). The FA methyl esters were identified and calibrated against commercial standards (Sigma Chemical Co.; Nu-Check-Prep, Elysian, MN, USA). The results were expressed as percentage (%) of total FAs in serum PL. Activity indices of SCD-16 and SCD-18 were determined calculating the ratios of 16:1n-7/16:0 and 18:1n-9/18:0, respectively, derived from serum PL.

### Statistical analysis

Statistical analyses of methylation data were performed with the software package R (version 3.1). Probes having the transcription start site (TSS) of SCD1 as closest TSS were extracted from the chip. Both upstream and downstream probes were included. Information about the exact distance to the TSS was extracted according to Price et al. [27]. A total of 19 SCD probes associated to SCD1 were identified and used in the analysis. Pairwise t-tests were performed to detect differences in DNA methylation between one-night NS and one-night TSD. Subsequently, we performed linear regression analyses regressing TSD induced changes in probe methylation against changes in the activity indices of SCD-16 (16:1n-7/16:0) and SCD-18 (18:ln-9/18:0) derived from serum PL. In all analyses the ratio of neutrophils to leukocytes was taken into account in order to prevent shifting monocyte subpopulations affecting the data (i.e. the regulation of immune and inflammatory responses). Wilcoxon signed-rank test (SPSS software, version 21.0; IBM Corporation, Somers, N.Y., USA) was used to compare intraindividual differences in 16:1n-7/16:0 and 18:ln-9/18:0 ratios before and after the two different sleep conditions. A *P*-value < 0.05 was considered statistically significant.

## Results

### Effect of one-night TSD on cytosine DNA methylation

**Fig. 1:**
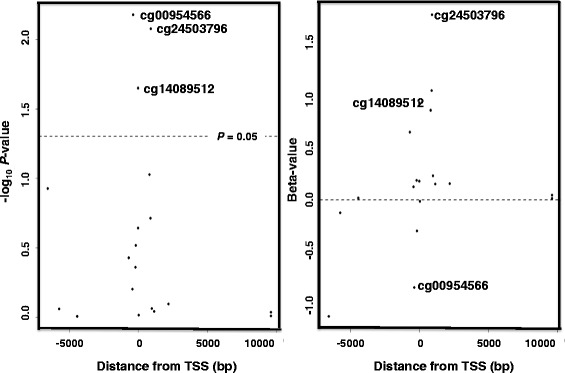
DNA methylation in relation to SCD1 position. Three of 19 SCD1 probes differ significantly in DNA methylation in dependency of sleep duration (one-night normal-sleep (NS) versus one-night total sleep deprivation (TSD); Left panel −log_10_ *P*-value). A similar trend towards hypermethylation was observed for probes close to the transcription start site (TSS) (Right panel Methylated beta-value)

Methylation of three SCD1 probes (cg00954566, cg24503796, cg14089512) close to the TSS was significantly different between one-night NS and one-night TSD in unadjusted analyses (−log_10_*P*-value > 1.3; Fig. 1: Left). Other probes, although not significant, displayed a similar trend towards hypermethylation after one-night TSD as in one-night NS. This was especially observed for CpG sites within 2000 bp up- or downstream of the TSS. Here, 11 of 14 probes showed a hypermethylation between 0.1 and 1.8 % after one-night TSD as in one-night NS (Fig. 1: Right).

### Association between cytosine DNA methylation and SCD activity indices

CpG sites found to be significantly changed in their methylation pattern after TSD were subsequently investigated in linear regression analyses regressing change of methylation to SCD activity indices. The methylation of three CpG sites was associated with the activity indices of SCD-16, namely cg19191454 (positively associated, (*P* < 0.001), cg23508052 and cg11311579 (negatively associated, *P* = 0.01 and *P* = 0.05, respectively) (Table 1). Furthermore, the probe cg15022173 was negatively associated (*P* = 0.02), and cg07649988 positively associated (*P* = 0.05) with the activity indices of SCD-18.

**Table 1:** Association between cytosine DNA methylation and SCD activity indices following one-night total sleep deprivation

Probe ID	Position	Relative TSS (bp)	Mean sleep % (sd)	Mean wake % (sd)	Slope	*P*-value	Slope	*P*-value
	Chr 10	NM_005063			SCD-16		SCD-18	
					(16:1n-7/16:0)		(18:1n-9/18:0)	
cg19191454	102106597	−175	4.4 (0.9)	4.2 (0.4)	0.016	**<0.001**	1.434	0.63
cg23508052	102106812	40	2.9 (0.5)	2.8 (0.5)	−0.003	**0.01**	−0.892	0.80
cg11311579	102106755	−17	0.8 (0.2)	1.0 (0.2)	−0.004	**0.05**	−6.887	0.38
cg01270221	102116383	9611	81.9 (1.3)	81.8 (1.2)	−0.092	0.07	−0.175	0.93
cg06400428	102107667	895	11.1 (3.5)	10.6 (3.6)	0.015	0.10	0.590	0.48
cg16744911	102108975	2203	82.0 (2.2)	82.4 (1.8)	0.025	0.19	0.213	0.87
cg03440556	102107758	986	27.6 (6.2)	26.6 (6.3)	0.039	0.25	−0.298	0.69
cg12714759	102102352	−4420	21.5 (3.9)	21.4 (3.8)	0.011	0.27	0.270	0.73
cg02237755	102107926	1154	83.1 (3.3)	83.1 (3.7)	0.048	0.55	−0.591	0.48
cg15022173	102101047	−5725	76.3 (2.2)	76.9 (2.4)	−0.420	0.56	−2.326	**0.02**
cg00653847	102100213	−6559	70.9 (4.1)	71.6 (3.5)	−0.025	0.61	−0.515	0.63
cg24503796	102107677	905	13.8 (3.2)	14.5 (2.9)	−0.018	0.65	0.766	0.53
cg07649988	102106577	−195	3.7 (0.7)	3.6 (0.8)	−0.019	0.72	8.026	**0.05**
cg14089512	102106758	−14	9.6 (1.1)	10.3 (1.2)	0.048	0.75	0.180	0.92
cg07230380	102106359	−413	0.7 (0.6)	0.9 (0.5)	0.004	0.92	1.160	0.75
cg18328965	102107585	813	11.0 (1.7)	10.8 (1.8)	0.221	0.93	1.902	0.14
cg00699831	102116399	9627	87.1 (1.1)	87.2 (1.3)	0.236	0.95	2.899	0.08
cg26351966	102106081	−691	18.2 (2.9)	18.7 (3.2)	0.017	0.96	0.050	0.97
cg00954566	102106406	−366	4.4 (0.9)	4.3 (0.9)	−0.002	0.97	0.474	0.90

The table shows selected SCD1 probes and SCD activity indices derived from serum phospholipids of young healthy men. Bold represents a statistically significant association at *P* < 0.05; software package R (version 3.1)

### Fatty acid composition and SCD activity indices

There were no significant changes of the levels of the SCD precursor and product, 16:0 and 16:1n-7, and 18:0 and 18:1n-9, respectively, in fasting serum PL following one-night NS. However, there were significant changes of individual FA levels, that resulted in elevated (*P* < 0.05) SCD-16 and SCD-18 activity indices derived from fasting serum PL following one-night TSD (Table 2).

**Table 2:** SCD activity indices before (day 1) and after (day 2) one-night normal-sleep (NS) or total sleep deprivation (TSD)

	NS (*n* = 12)		TSD (*n* = 14)	
	Day 1	Day 2	Day 1	Day 2
Fatty acids (% of total FAs)				
16:0	26.78 ± 0.44	26.80 ± 0.35	26.58 ± 0.37	27.00 ± 0.24^a ^
16:1n-7	0.41 ± 0.04	0.43 ± 0.04	0.37 ± 0.03	0.40 ± 0.02^a ^
18:0	12.78 ± 0.34	12.00 ± 0.51	12.55 ± 0.22	12.35 ± 0.16^a ^
18:1n-9	9.40 ± 0.33	9.54 ± 0.33	9.51 ± 0.25	9.74 ± 018^a^
Activity indices				
SCD16	0.0150 ± 0.0014	0.0159 ± 0.0014	0.0140 ± 0.0009	0.0147 ± 0.0009^a^
(16:1n-7/16:0)				
SCD18	0.7641 ± 0.0351	0.8088 ± 0.0367	0.7591 ± 0.0198	0.7904 ± 0.0170^a^
(18:1n-9/18:0)				

The table shows the SCD activity indices derived from serum phospholipids of young healthy men

Data are expressed as mean ± SEM

^a^ *P* < 0.05 compared with day 1, Wilcoxon signed-rank test

## Discussion

We demonstrate, for the first time to our knowledge that one-night TSD is significantly associated with increased methylation in the 5’ prime region of the SCD1 gene. Importantly, we show that one-night TSD related methylation changes of SCD1 are significantly associated with changes in SCD-16 and SCD-18 activity indices derived from fasting serum PL followed by one-night TSD in young normal-weight healthy men, thus, describing a novel regulatory pathway by which TSD may influence homeostasis of body fat.

Recently it was demonstrated in the subjects of the present study, that a one-night TSD alters the epigenetic and transcriptional profile of core circadian clock genes in key metabolic tissues [12], thus demonstrating TSD systematic changes in methylation of functionally important gene networks. Increased DNA methylation has been, in many cases, associated with a suppressed gene expression [28]. We show that one night TSD is associated with increased methylation in the 5’ prime region of the SCD1, which is in line with a recent study showing that methylation shifts are able to suppress or enhance gene expression, suggesting two different mechanisms of DNA methylation-dependent gene regulation [29]. Our findings that SCD1 methylation differs between one-night NS and one-night TSD strongly support the hypothesis that TSD is connected to epigenetic shifts that might have impact on lipid metabolism [30].

It is known from several experimental studies that changes in the activity of SCD indices estimated from plasma or tissues are accompanied by simultaneous changes in the transcriptional level of the SCD1 [31, 32]. The regulation of SCD1 gene is of considerable physiological importance, as a high SCD activity has been implicated in a wide range of disorders including diabetes, atherosclerosis, cancer, and obesity [14, 33, 34]. Thus, our observation, that one-night TSD is associated with increased methylation of the SCD1, may allow the speculation that TSD has the ability to contribute to the mentioned metabolic diseases via changes in the methylation pattern of genes, such as SCD1.

Short sleep duration may increase obesity risk by causing small changes in eating patterns that cumulatively alter energy balance [35, 36]. Several studies have indicated associations between insufficient sleep and alterations in circulating hormones involved in feeding behavior, glucose metabolism, hunger, and appetite, which are probably involved in the development of metabolic disorders, such as obesity and diabetes [9, 10]. Recent study has shown that higher habitual sleep variability, but not habitual sleep duration, is significantly associated with abdominal obesity in adolescents, which can be partially explained by increased caloric intake, especially from carbohydrates [37]. The precise mechanism through which the brain regulates changes in hormone release with sleep deprivation is unknown, but one possibility is increased sympathetic nervous system activity [38].

Several studies have reported that diet affects the FA composition of serum PL [39–41]. Our observation indicates similar dietary habits and lifestyle factors such as cigarette smoking and alcohol consumption among the participants, since there was no significant difference found in FA levels of non-fasting serum PL between the two sleep conditions separated by 4 weeks (day 1). On the other hand, there is no general agreement which blood lipid fraction to use regarding the assessment of hepatic SCD activity indices. The majority of PL synthesis occurs in the endoplasmic reticulum of the liver, where PL associates with other lipids and proteins resulting in lipoproteins that are released into the bloodstream. Thus, we assume that in fasting blood the SCD-16 and SCD-18 activity indices derived from serum PL may mainly reflect hepatic SCD-1 activity. We observed significantly elevated SCD-16 and SCD-18 activity indices derived from fasting serum PL following one-night TSD and before a standardized breakfast. This strengthens the hypothesis that shortened sleep is an additional link to the dysregulation in energy metabolism that might impact on lipid metabolism and weight gain [30, 42].

This study is limited by its relatively small sample size of healthy individuals, which may preclude enough power to assess the interaction between SCD1 methylation and sleep deprivation. Another limitation of our study is that the methylation of SCD1 was measured only at a single time point, i.e., under fasting condition in the morning following each sleep intervention. Thus, conclusions about the causality have to be made carefully. However, our observations allow to suggesting a role of sleep duration and quality in regulating SCD1 expression and development of obesity.

## Conclusions

Our study results indicate that one-night TSD modifies SCD1 methylation, FA levels of serum PL, and, thereby, changes in SCD-16 and SCD-18 activity indices. The exact mechanism how sleep restriction is implicated in SCD1 expression and development of obesity warrants further investigation.
